# A New Modification of Temporary Suture-Holding Technique for Transcatheter Repair of Sinus Venosus Atrial Septal Defect

**DOI:** 10.1007/s00246-025-04154-3

**Published:** 2026-01-21

**Authors:** Ihsan A. Elhalabi, Ziyad M. Hijazi

**Affiliations:** Department of Cardiovascular Diseases, Sidra Medicine, Doha, Qatar

**Keywords:** Sinus venosus atrial septal defect, Transcatheter SVASD closure, Suture-holding technique, Covered stent

## Abstract

Transcatheter repair of superior sinus venosus atrial septal defect (SVASD) and associated partial anomalous pulmonary venous return (PAPVR) using a covered stent has evolved as a less invasive alternative to surgery. Precise control of stent position remains a key technical challenge during the procedure. Previously, our group has reported on the suture technique with improved outcome. Here, we further report on an enhanced/modified suture technique. We report on a 14-year young boy with a superior SVASD associated with PAPVR of the right middle and upper pulmonary veins who underwent a successful transcatheter repair of the defect using a covered stent with a modification of the previously described novel temporary suture-holding technique by our group. The new modified technique (the holding-wire technique-HWT) uses a 150 cm, 0.018” Terumo® guidewire positioned in the outermost strut of the stent and both ends of the wire are exteriorized from the internal jugular vein to hold the stent temporarily instead of the suture. This allowed precise control and better grip of the attached stent, as well as visualizing the wire on fluoroscopy throughout the procedure. An 80-mm covered stent was deployed and anchored with a 28-mm bare-metal stent proximally, achieving complete exclusion of the atrial communication and rerouted the anomalous vein flow to the left atrium without any obstruction. This simple modification enhances procedural control and safety, and may broaden the clinical applicability of transcatheter SVASD closure

## Introduction

Sinus venosus atrial septal defect (SVASD) and associated partial anomalous pulmonary venous return (PAPVR) traditionally required surgical repair. In the last decade, development and advancements in transcatheter techniques using covered stents have made transcatheter repair a reliable alternative to exclude the atrial communication and reroute the anomalous pulmonary veins drainage in suitable defects [1, 2, 3]. The previously described suture-holding technique [4] by our group, provided improved control and minimized stent migration (Achilles heal of this procedure) during the balloon expansion of the stent. Building on this concept we further modified this technique (suture technique) using a radiopaque guidewire as a “holding-wire” to provide secure traction and superior visualization. This modification (holding-wire technique-HWT) aims to provide radiopacity, greater strength reducing breakage of the suture and provides more length outside the body to control the stent better particularly in older and taller population when additional length was not possible due to the limited availability of 100 cm sutures.

## Case Presentation

**Fig. 1:**
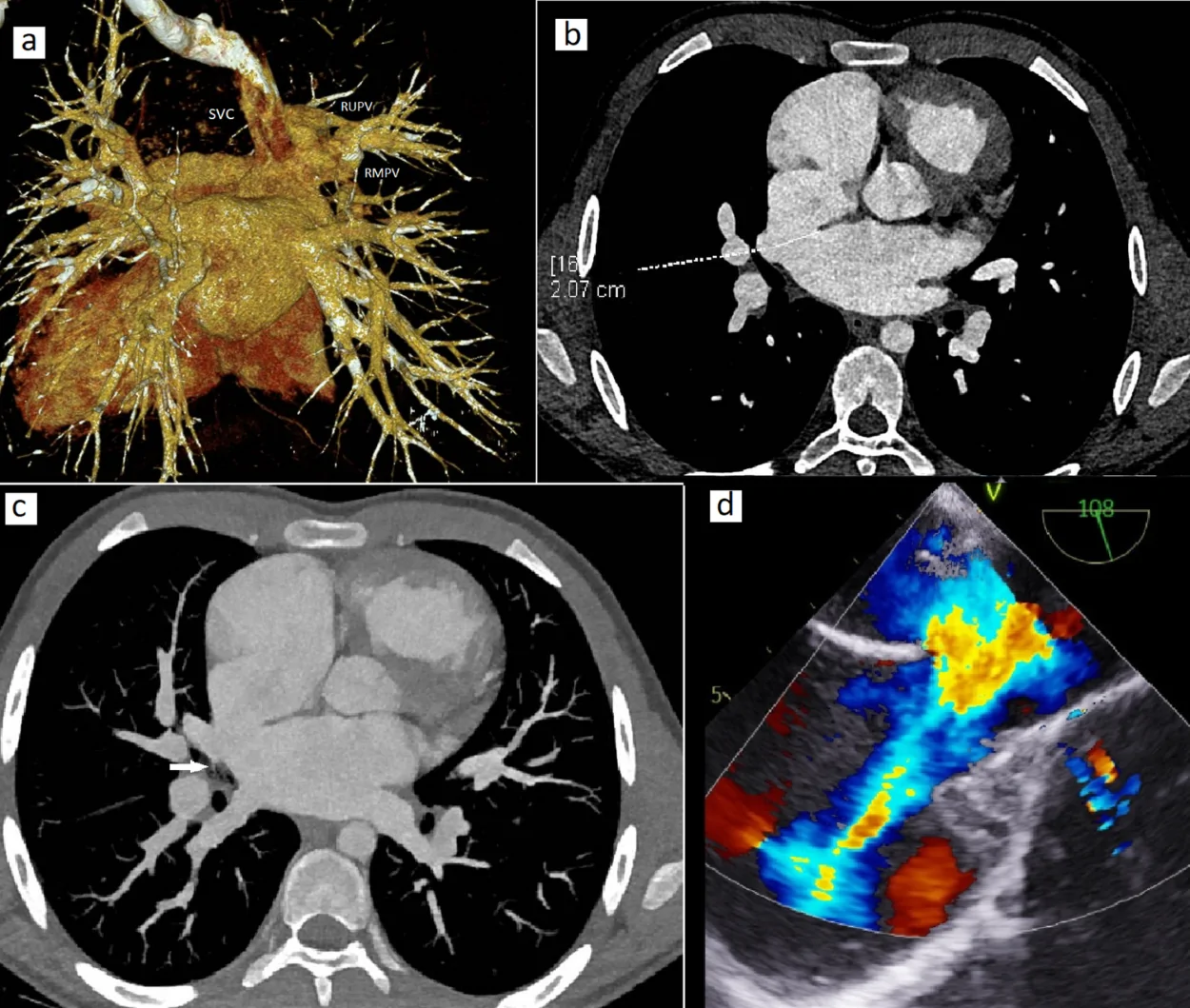
Pre-procedural CT scan and transesophageal echocardiography (TEE). **a**: 3D reconstruction showing the SVC–RA junction with connection of the anomalous right middle and lower pulmonary veins. **b **and** c**: Cross-sectional views of the defect measuring approximately 21 mm, with anomalous pulmonary venous drainage into the RA (white arrow). **d**: TEE image demonstrating a left-to-right shunt across the superior sinus venosus ASD

A 14-year young asymptomatic boy was referred to us for catheter repair of his SVASD associated with partial anomalous pulmonary venous drainage (PAPVR) of his right middle (RMPV) and right upper pulmonary veins (RUPV) that drained at the superior vena cava (SVC) and right atrial (RA) junction. His diagnosis was confirmed by transthoracic echocardiography (TTE), transesophageal echocardiography (TEE) and computed tomography (CT) imaging (Fig. 1). His electrocardiogram revealed normal sinus rhythm, right axis deviation and rsr’ in lead V1 confirming right ventricular enlargement. TEE revealed the ASD to be approximately 21 mm in diameter. The distance from the innominate to the inferior margin of the SVASD measured on CT scan was approximately 65 mm. The cardiac CT scan confirmed the diagnosis of the SVASD with PAPVR of the RUPV and RMPV to SVC-RA junction (Fig. 1a-c) and ruled out other abnormalities. The case was reviewed in our multidisciplinary cardiac center meeting and transcatheter repair of the SVASD using a covered stent was recommended.

## Procedure

**Fig. 2:**
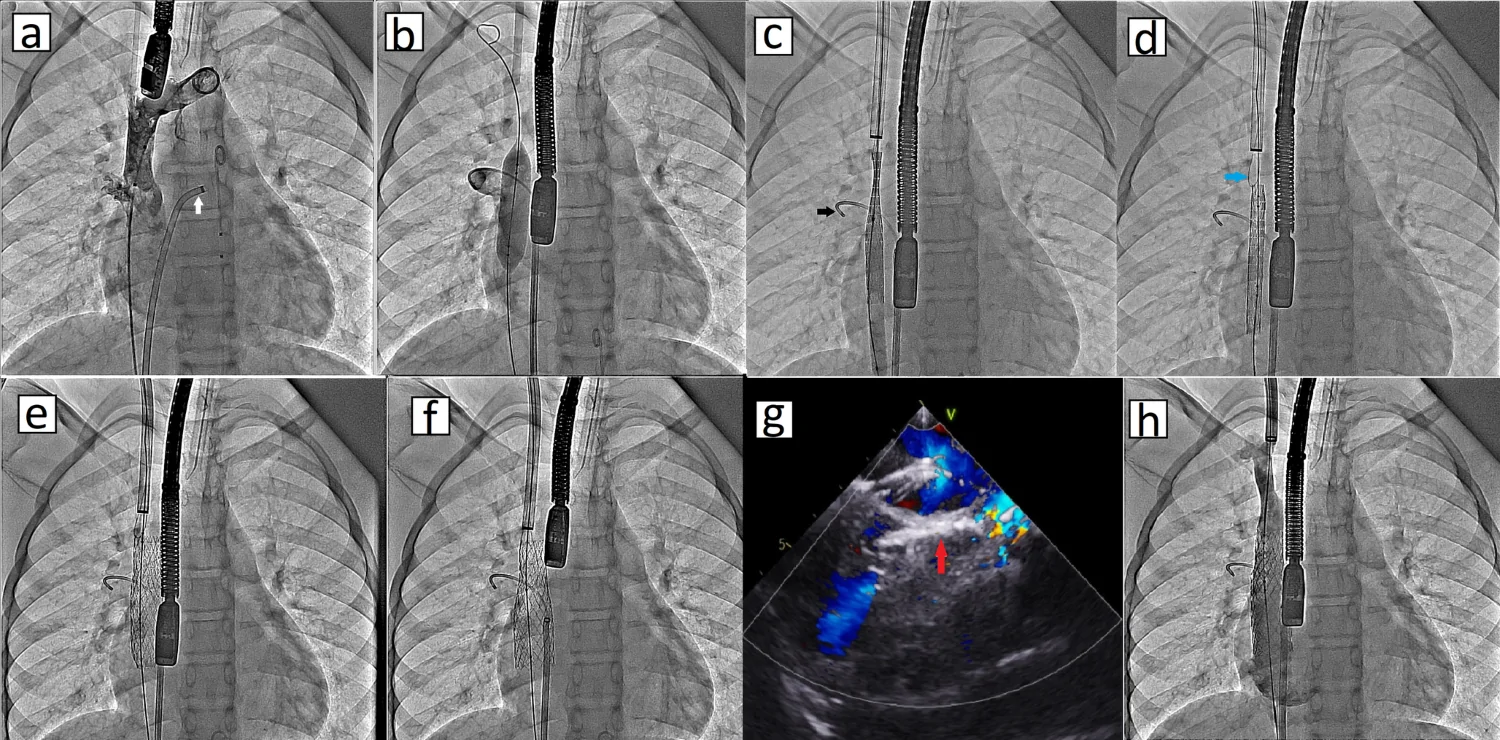
Step-by-step procedural angiography and TEE images. **a**: Innominate vein injection opacifying the SVC, RA, and anomalous pulmonary venous connection to the SVC. The white arrow shows the catheter in the LA following transseptal puncture. **b**: Balloon inflation in the SVC demonstrating redirection of anomalous pulmonary venous flow into the LA. **c**: An 80-mm Optimus-CVS covered XXL stent held by a 150-cm 0.018” Terumo wire. The stent was initially mounted on a 10 mm × 8 cm Mustang balloon and advanced. A catheter was maintained in the anomalous vein for pressure monitoring after crossing into the LA (black arrow). **d**: The stent was inflated to 10 mm, the balloon was removed, and the stent was positioned to cover the posterior margin of the defect while being held by the wire (blue arrow). **e**: The stent was further expanded using an 18-mm BiB balloon to achieve apposition to the SVC wall f: The distal portion of the stent was inflated twice using a 25-mm Tyshak balloon to ensure elimination of any residual shunt. **g**: TEE image showing the stent in position within the RA with no residual shunt. **h**: To ensure stability of the covered stent, a 28-mm bare-metal stent was deployed proximally with approximately 50% overlap. Final angiography confirmed secure positioning after removal of the holding wire

The procedure was performed under general endotracheal anesthesia and continuous TEE guidance. Ultrasound guided vascular access was obtained in the right femoral vein (RFV), left femoral vein (LFV) and artery (LFA) and right internal jugular vein (RIJ). Right and left heart catheterization was performed. His Qp: Qs was calculated at 2.5:1 and his pulmonary artery pressure was normal. RFV access was eventually upgraded to 16Fr DrySeal sheath [Gore Medical, Newark, DE], RIJV access was also upgraded to 18Fr sheath and the LFV access was upgraded to 8Fr sheath for the transeptal puncture. The transeptal puncture was performed using the Brockenbrough needle [Abbott Medical, Santa Clara, CA] and a 5 French JR catheter was left in the confluence of the anomalous RMPV & RUPV during the entire procedure to monitor pressure and for contrast injections. Angiography in the SVC and the anomalous pulmonary veins was performed. This revealed the diameter of the SVC above the entrance of the anomalous pulmonary vein to be 14–16 mm and below at the RA junction to be almost 19 mm (Fig. 2a, b).

**Fig. 3:**
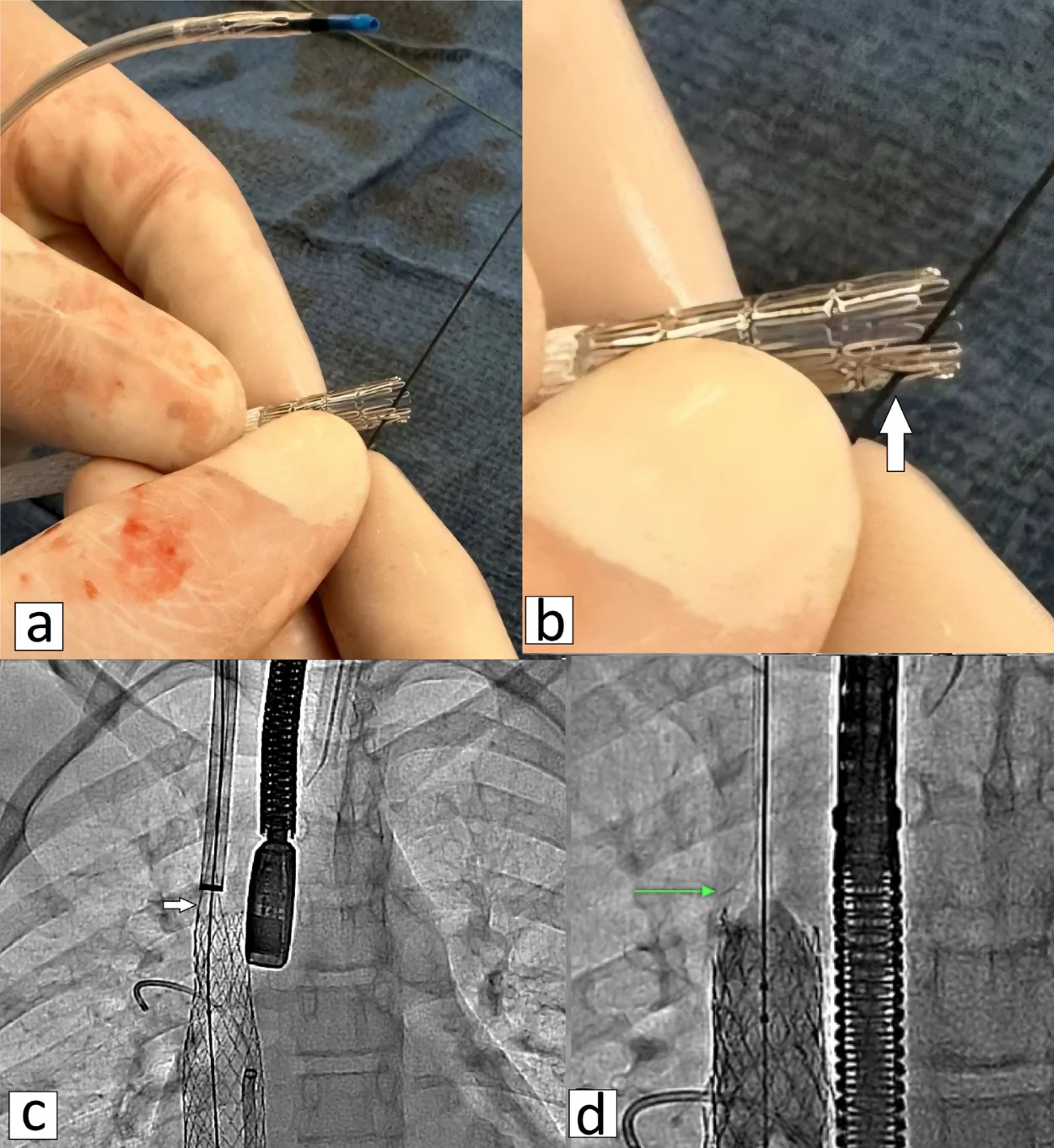
Holding-Wire technique. **a**: The 80-mm Optimus-CVS covered XXL stent secured using a 150-cm 0.018” Terumo wire passed through the outermost strut. **b**: Zoomed image demonstrating the wire position (white arrow). **c**: The wire was visualized fluoroscopically throughout the procedure (white arrow). **d**: Removal of the wire after successful stent positioning (green arrow)

An 80 mm Optimus-CVS covered XXL stent [AndraTec, Germany, Koblenz] was held by a 150 cm 0.018” Terumo wire [Terumo, Japan, Tokyo] using the outermost strut (Fig. 3a, b) of the stent. This wire held the stent proximally while it was crimped on the balloon, leaving both ends of the wire holding the stent outside the body, thus providing control of the position of the stent once inside the body. The stent was then initially mounted on a 76 cm long, 10 mm x 8 cm Mustang balloon [Boston Scientific, Marlborough, MA] and advanced into the DrySeal sheath from the RIJ vein, with the wire holding it from outside the body Fig. 2c. The stent then was inflated and positioned to cover both the inferior margin of the defect as confirmed by TEE and fluoroscopy. The Mustang balloon was removed, leaving the stent in position using the wire holding it Fig. 2d. The operator has the ability to pull the stent proximally in the SVC by pulling the wire holding the stent, or if the stent is not covering the defect in the atrial septum, the stent can be dragged down by a balloon, yet still fully controlled by the wire holding the stent. Then an 18 mm x 5.5 cm BiB balloon [NuMED Inc, Hopkinton, NY] was advanced from the RIJ sheath until it reached the proximal part of the stent. The balloon was inflated to appose the stent to the wall of the SVC. The balloon was further advanced to the section of the stent in the RA and was inflated Fig. 2e. Repeat angiography in the SVC revealed that the stent was fully apposed to the wall of the SVC. TEE revealed presence of residual shunt. Injection in the anomalous PV revealed no obstruction to flow with normal pressure similar to that of the left atrial pressure transduced via the transseptal sheath. The BiB balloon was removed and a 25 mm x 4 cm Tyshak Balloon [NuMED Inc, Hopkinton, NY] was introduced from the RIJ and advanced to the distal part of the stent in the RA. Under TEE guidance this balloon was inflated couple of times until cessation of flow from LA to RA was confirmed (Fig. 2f). Then to ensure covered stent stability and eliminate risk of migration after removal of the wire holding the stent, a 28 mm bare metal AndraTec [AndraTec, Germany, Koblenz] stent was mounted on a 16 mm x3.5 cm BiB balloon and inflated 50% inside the stent and the other 50% inside the SVC just distal to the innominate vein. Repeat angiography confirmed good stents position and TEE revealed complete closure of the SVASD and the anomalous vein was draining to the LA (Fig. 2g, h) with no gradient in pressure between the anomalous pulmonary vein and the LA. Final step was removal of the 0.018” Terumo wire holding the stent under fluoroscopy monitoring by pulling the stiff end of the wire first (Fig. 3c, d). At the end of procedure hemostasis was achieved by using the Proglide [Abbott Medical, Santa Clara, CA] in RFV and RIJ (preclosure was done at the beginning of the procedure) and by direct compression on left FV and LFA.

**Fig. 4:**
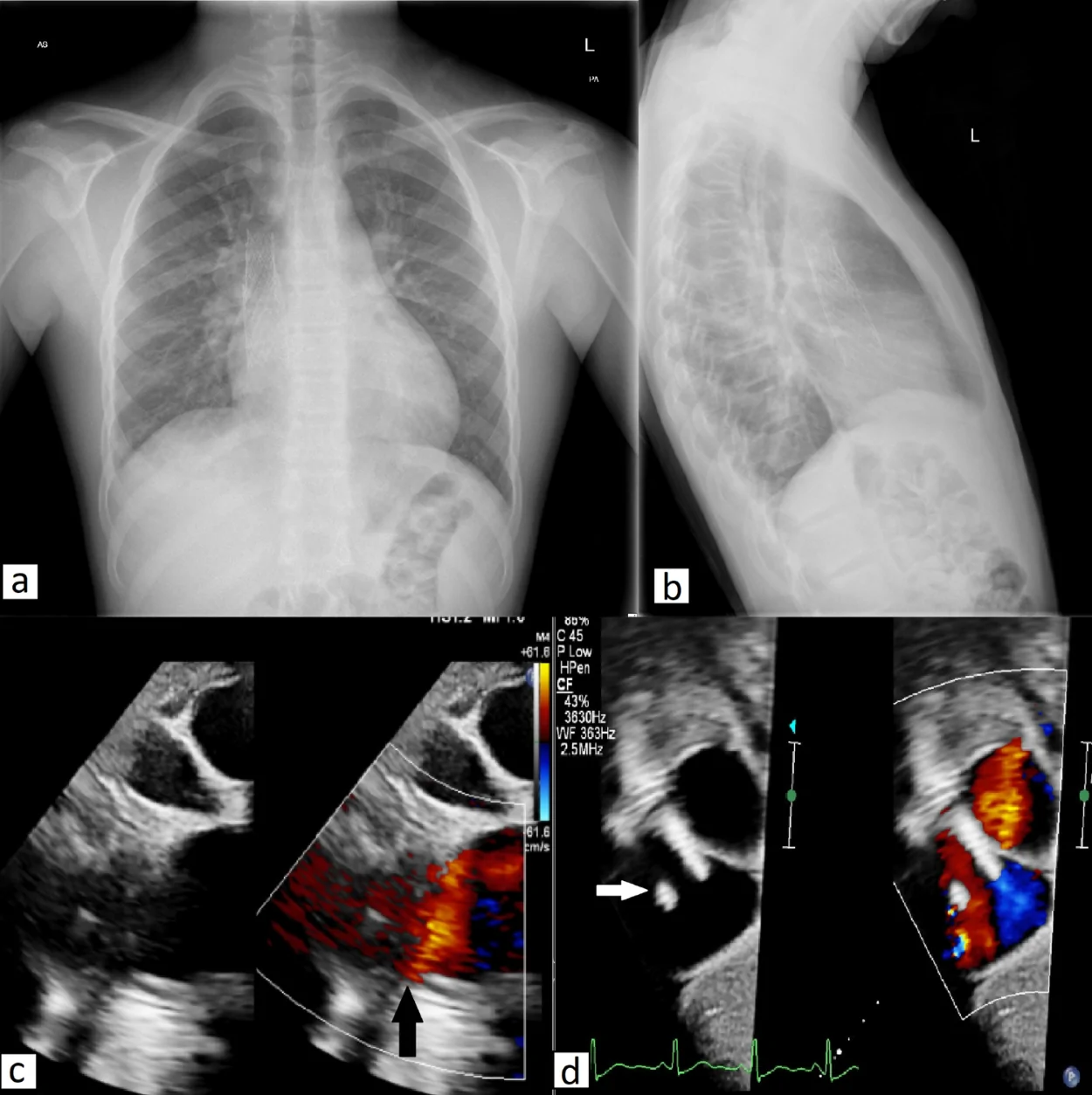
Post-procedural follow-up. **a **and** b**: Anteroposterior and lateral chest X-rays obtained one day post-procedure showing the stent in stable position. **c **and** d**: Echocardiography one day post-procedure demonstrating laminar flow from the right pulmonary veins into the LA (black arrow), with the stent well-positioned and no residual shunt (white arrow)

The patient recovered overnight and the following morning had a chest Xray, TTE (Fig. 4) and EKG. These revealed that the stents were in good position, no residual shunt and patient was in normal sinus rhythm. He was discharged home on 75 mg aspirin daily for one year and 75 mg Clopidogrel daily for 2 months.

## Discussion

Since the introduction of transcatheter closure of SVASD in 2011 by Abdullah HAM et al. [1] interventionists focused on improving the procedural techniques, applicability and to minimize complications. Stent caudal embolization and pulmonary venous return obstruction are still among the most faced complications. Embolization risk was significantly reduced with the introduction of the suture-holding technique by our group. There are other techniques employed to minimize the risk of embolization. These include the use of an initial landing-zone stent before deploying the covered stent [3]. “Sandwich technique” by having a second bare metal stent after deploying the covered stent over a landing zone stent at the top end to sandwich both stents [5]. Using a modified suture control technique as reported by Haddad RN et al., [6] with the covered stent hand-mounted on large balloon catheter and then the stent cell was crossed with a suture to control. Stents suturing technique was reported by Tan W et al., [7] by suturing two 60 mm and 50 mm covered stents using a suture with overlap prior to the procedure.

In this paper, we further report on a new modification by using a guidewire to hold the stent instead of a suture. This HWT provides more mechanical strength, resisting stretching or breakage even during high tension, unlike sutures that might break, a strength that ensures sustained control during manipulation and inflation of the covered stent. Moreover, the guidewire can be visualized during fluoroscopy being radiopaque, providing real-time visualization with confirmation of wire tension and stent orientation. Guidewires usually have multiple lengths and give more flexibility when additional length is needed. Together, these advantages make using a guidewire as a holding mechanism an evolution to the traditional suture-holding technique – more visible, durable and versatile – while preserving the safety advantage of mechanical control of the stent. This modification can be easily adopted using standard cath lab equipment. Future studies on its use will determine its effectiveness and improvements in procedural outcomes. Of note this modification and the original suture technique require deployment of the stent from the internal jugular vein so that the suture or the wire in this case are exteriorized from the internal jugular and the operator has full control of the position of the stent. The operator can pull the stent up prior to full expansion by pulling on the wire or if the stent needs to be positioned lower, a balloon inside the stent can drag it down.

## Conclusion

The radiopaque, high-strength, and longer holding-wire modification represents an improvement on the suture -holding technique for transcatheter repair of SVASD. This technique provides continuous mechanical control, superior fluoroscopic visibility and length flexibility and will enhance both safety and feasibility of a complex transcatheter intervention.

## Data Availability

No datasets were generated or analysed during the current study.
